# Genetic Associations with Plasma B12, B6, and Folate Levels in an Ischemic Stroke Population from the Vitamin Intervention for Stroke Prevention (VISP) Trial

**DOI:** 10.3389/fpubh.2014.00112

**Published:** 2014-08-06

**Authors:** Keith L. Keene, Wei-Min Chen, Fang Chen, Stephen R. Williams, Stacey D. Elkhatib, Fang-Chi Hsu, Josyf C. Mychaleckyj, Kimberly F. Doheny, Elizabeth W. Pugh, Hua Ling, Cathy C. Laurie, Stephanie M. Gogarten, Ebony B. Madden, Bradford B. Worrall, Michele M. Sale

**Affiliations:** ^1^Center for Public Health Genomics, University of Virginia, Charlottesville, VA, USA; ^2^Department of Biology, Center for Health Disparities, East Carolina University, Greenville, NC, USA; ^3^Department of Public Health Sciences, University of Virginia, Charlottesville, VA, USA; ^4^Department of Biostatistical Sciences, Wake Forest School of Medicine, Winston Salem, NC, USA; ^5^Center for Inherited Disease Research, Johns Hopkins University School of Medicine, Baltimore, MD, USA; ^6^Department of Biostatistics, University of Washington, Seattle, WA, USA; ^7^National Human Genome Research Institute, National Institutes of Health, Bethesda, MD, USA; ^8^Department of Neurology, University of Virginia, Charlottesville, VA, USA; ^9^Department of Biochemistry & Molecular Genetics, University of Virginia, Charlottesville, VA, USA

**Keywords:** VISP, association, GWAS, one-carbon metabolism, B12, B6, folate

## Abstract

**Background:** B vitamins play an important role in homocysteine metabolism, with vitamin deficiencies resulting in increased levels of homocysteine and increased risk for stroke. We performed a genome-wide association study (GWAS) in 2,100 stroke patients from the Vitamin Intervention for Stroke Prevention (VISP) trial, a clinical trial designed to determine whether the daily intake of high-dose folic acid, vitamins B_6_, and B_12_ reduce recurrent cerebral infarction.

**Methods:** Extensive quality control (QC) measures resulted in a total of 737,081 SNPs for analysis. Genome-wide association analyses for baseline quantitative measures of folate, Vitamins B_12_, and B_6_ were completed using linear regression approaches, implemented in PLINK.

**Results:** Six associations met or exceeded genome-wide significance (*P* ≤ 5 × 10^−08^). For baseline Vitamin B_12_, the strongest association was observed with a non-synonymous SNP (nsSNP) located in the *CUBN* gene (*P* = 1.76 × 10^−13^). Two additional *CUBN* intronic SNPs demonstrated strong associations with B_12_ (*P* = 2.92 × 10^−10^ and 4.11 × 10^−10^), while a second nsSNP, located in the *TCN1* gene, also reached genome-wide significance (*P* = 5.14 × 10^−11^). For baseline measures of Vitamin B_6_, we identified genome-wide significant associations for SNPs at the *ALPL* locus (rs1697421; *P* = 7.06 × 10^−10^ and rs1780316; *P* = 2.25 × 10^−08^). In addition to the six genome-wide significant associations, nine SNPs (two for Vitamin B_6_, six for Vitamin B_12_, and one for folate measures) provided suggestive evidence for association (*P* ≤ 10^−07^).

**Conclusion:** Our GWAS study has identified six genome-wide significant associations, nine suggestive associations, and successfully replicated 5 of 16 SNPs previously reported to be associated with measures of B vitamins. The six genome-wide significant associations are located in gene regions that have shown previous associations with measures of B vitamins; however, four of the nine suggestive associations represent novel finding and warrant further investigation in additional populations.

## Introduction

The B vitamins constitute a group of water-soluble vitamins that play an important role in human health and cellular functions including growth and development (1). Vitamins B_6_ (pyridioxine), B_9_ (folic acid or folate), and B_12_ (cobalamin) have garnered extensive attention for their putative impacts on human health and diseases, ranging from cardiovascular disease and stroke to neurocognitive function and depression. Specifically, these B vitamins are critical for the maintenance of red blood cells (2), components of the nervous (3), and immune systems (4). Vitamin B_6_ deficiency, most common in the elderly, has been associated with conditions such as anemia, and neurological abnormalities such as depression, cognitive dysfunction, and neuropathy (5). Vitamin B_12_ deficiency can result in irreversible brain and nervous system damage and may be responsible for common symptoms such as fatigue and poor memory (6, 7). Folate (Vitamin B_9_) is critical for fetal growth and brain development, therefore folate deficiencies during pregnancy can result in neural tube defects in babies (8). In addition, Vitamins B_6_, B_9_, and B_12_ serve as important factors in homocysteine metabolism, with vitamin deficiencies resulting in increased levels of homocysteine (9, 10). Although controversial, elevated homocysteine levels are thought to increase risk for stroke (11) and vascular disease (12, 13).

Multiple factors contribute to variations in B vitamin levels in humans. A balanced diet is one approach to help minimize the detrimental effects of B vitamin deficiency. In January 1998, the United States Food and Drug Administration required manufacturers to fortify bread and grain products with folic acid to help prevent neural tube defects due to Vitamin B_9_ deficiency. These efforts have proven somewhat successful, with estimates from the 2002–2006 National Health and Nutrition Examination Survey (NHANES) reporting that most Americans are receiving adequate amounts of folate (14). In contrast, for Vitamin B_12_, data suggest that 5–15% of elderly patients are Vitamin B_12_ deficient, including data from the Centers for Disease Control and Prevention (CDC) and the NHANES study (15–17). Poor dietary intake, malabsorption from food, and genetic predisposition may all cause vitamin deficiencies. Polymorphisms in genes involved in B vitamin metabolism and processing, transport, absorption, and excretion are logical candidate genes that can influence B vitamin levels. Two such examples include human conditions Imerslund–Grasbeck syndrome (IGS) and megaloblastic anemia-1. IGS, a rare autosomal recessive disorder caused by mutations in cubilin (*CUBN*) and/or amnionless (*AMN*), was first characterized in the 1960s (18, 19) and results in megaloblastic anemia during childhood as a result of selective malabsorption of Vitamin B_12_. Additionally, genetic variants in the *CUBN* and *AMN* genes are responsible for the Finnish and Norwegian types of megaloblastic anemia-1, respectively (20, 21).

Understanding the genetic factors contributing to vitamin deficiencies offers opportunities for screening and identification of high-risk individuals before the presentation of any clinical manifestations. To date, several large-scale genome-wide association studies (GWAS) testing for association with Vitamin B_6_, B_12_, and folate have been published, resulting in more than 10 confirmed loci for these traits (22–25). Our group has conducted a GWAS for Vitamin B_6_, B_12_, and folate in an effort both to identify novel associations and replicate previously reported associations for these traits in a population of ischemic stroke patients from the Vitamin Intervention for Stroke Prevention (VISP) clinical trial, an NIH-funded, multi-center, double-blind, randomized, controlled clinical trial designed to determine whether the daily intake of high-dose folic acid, Vitamins B_6_, and B_12_ reduced recurrent cerebral infarction and a combined vascular endpoint. Unlike the previous GWAS, the VISP study population represents an ethnically diverse population of older patients that present with elevated baseline homocysteine levels in the top quartile, have suffered a stroke, and thus, more closely represent the elderly population that is most prone to vitamin B deficiency and stroke.

## Materials and Methods

### Subjects

The VISP trial was a multi-center, double-blind, randomized, and controlled clinical trial that enrolled patients aged 35 or older with homocysteine levels above the 25th percentile at screening and a non-disabling cerebral infarction (NDCI) within 120 days of randomization (26, 27). NDCI was defined as an ischemic brain infarction not due to embolism from a cardiac source, characterized by the sudden onset of a neurological deficit. The deficit must have persisted for at least 24 h, or if not, an infarction in the part of the brain corresponding to the symptoms must have been demonstrated by CT or MRI imaging. The trial was designed to determine if daily intake of a multivitamin tablet with high-dose folic acid, vitamin B_6_, and vitamin B_12_ reduced recurrent cerebral infarction and non-fatal myocardial infarction (MI) or mortality. Subjects were randomly assigned to receive daily doses of the high-dose formulation (*n* = 1,827), containing 25 mg pyridoxine (B_6_), 0.4 mg cobalamin (B_12_), and 2.5 mg folic acid; or the low-dose formulation (*n* = 1,853), containing 200 μg pyridoxine, 6 μg cobalamin, and 20 μg folic acid. Enrollment in VISP began in August 1997, and was completed in December 2002, with 3,680 participants enrolled from 55 clinic sites across the U.S. and Canada and one site in Scotland. All human research was approved by the relevant institutional review boards (IRBs), and conducted according to the Declaration of Helsinki. The VISP study protocol was approved by the IRBs of Wake Forest School of Medicine (coordinating center) and the University of North Carolina at Chapel Hill School of Medicine (statistical center). The local IRB for each of the individual recruiting sites approved the VISP protocol and all participants provided written, informed consent. VISP data analysis by the Genomics and Randomized Trial Network (GARNET) was approved by University of Virginia School of Medicine IRB.

### Genome-wide association study in VISP

A subset of VISP participants provided consent for inclusion in genetic studies. These participants were included in the GWAS component of VISP, supported by the National Human Genome Research Institute (NHGRI), Grant U01 HG005160, as part of the Genomics and Randomized Trials Network (GARNET); dbGaP Study Accession: phs000343.v3.p1. Samples were genotyped at the Johns Hopkins Center for Inherited Disease Research (CIDR), using the Illumina HumanOmni1-Quad_v1-0_B BeadChip (Illumina, San Diego, CA, USA). Individuals were excluded if they were unexpected duplicates or had gender discrepancies. A total of 2,100 individuals were included in the final genetic analyses; summary statistics are provided in Table 1. These subjects consisted of 1,725 individuals of European descent, 258 individuals of African descent, and 117 individuals classified as others.

**Table 1:** **Demographic summary statistics**.

Number of individuals (EA/AA/other)	2100 (1725/258/117)
Age (years)
Mean ± SD	67.2 ± 10.8
Range	35–89
% Female participants (N)	37.4 (785)
Current smokers (%)	15.6
Hypertension (%)	71.0
Diabetes mellitus (%)	27.1
**B Vitamin baseline measures**
Vitamin B_6_ (pm/mL) ± SD	42.45 ± 37.38
Median	33.49
Vitamin B_12_ (pg/mL) ± SD	358.79 ± 181.91
Median	326
Folate (ng/mL) ± SD	25.86 ± 15.91
Median	22.67

### Biomarker measurements in VISP

As previously described (28), basal levels of folate and Vitamin B_12_ were determined by the central laboratory at Oregon Regional Primate Research Center using single radioassays of folate and Vitamin B_12_ (Bio Rad Quantaphase II, Bio Rad Diagnostics, Hercules, CA, USA). For measures of Vitamin B_6_, EDTA plasma samples were analyzed using a commercially available method for plasma Pyridoxine 5′ Phosphate (ALPCO Inc Windham, NH, USA). The principle of the assay is as follows: ^3^H-tyrosine is decarboxylated by the vitamin B_6_ dependent enzyme tyrosine apodecarboxlase to ^3^H-tyramine. The activity of tyrosine apodecarboxlase is quantitatively dependent on the amount of PLP present in the reaction mixture. The ^3^H-tyramine thus produced is selectively extracted into the scintillation cocktail and can be measured by liquid scintillation counting. The excess ^3^H-tyrosine remains in the aqueous phase and is not measured.

### Statistical analyses

Extensive quality control (QC) measures were performed, resulting in a total of 737,081 SNPs for analysis. QC measures included filtering SNPs based on missing call rate, Mendelian errors in control trios, deviation from Hardy–Weinberg equilibrium in controls, discordant calls in duplicate samples, sex differences in allele frequency or heterozygosity, and minor allele frequency (MAF) (29). Briefly, samples were clustered and genotypes determined using GenomeStudio (version 2010.2). For initial QC, SNPs meeting one or more of the following criteria were excluded: call rate <85%, more than one replicate HapMap error, or cluster separation <0.2. Genotype calls for individual chromosomes in samples with large chromosomal anomalies (>10 Mb) or missing call rate >5% were filtered out. Furthermore, samples with overall missing call rates >5% and SNPs with call rates <95% and Hardy–Weinberg *P*-values ≤10^−4^ were excluded from subsequent analyses. Multidimensional Scaling (MDS), utilizing the software KING (30), was performed to address confounders due to population substructure. Genome-wide association analyses for baseline quantitative measures of folate, and Vitamins B_12_ and B_6_ were performed using linear regression approaches, assuming an additive model, as implemented in PLINK; using age, sex, and the top 10 principal components as covariates. Inverse normal transformation was performed for each of the quantitative traits, prior to analysis. Inverse normal transformations were used to maintain ranks of the trait for each individual and minimize the impact of outliers while also allowing for sufficient power. Regression coefficients (beta), coefficient T-statistic (STAT), and *P*-values (asymptotic *P*-value for *T*-statistic) were calculated for the tested (minor) allele. The proportion of total variance explained (*h*^2^) was calculated as *h*^2^ = Beta^2^ × 2 × MAF × (1-MAF). Our GWAS scan results showed no evidence for inflation (GC lambda ≤ 1.013 in all scans.).

## Results

**Figure 1:**
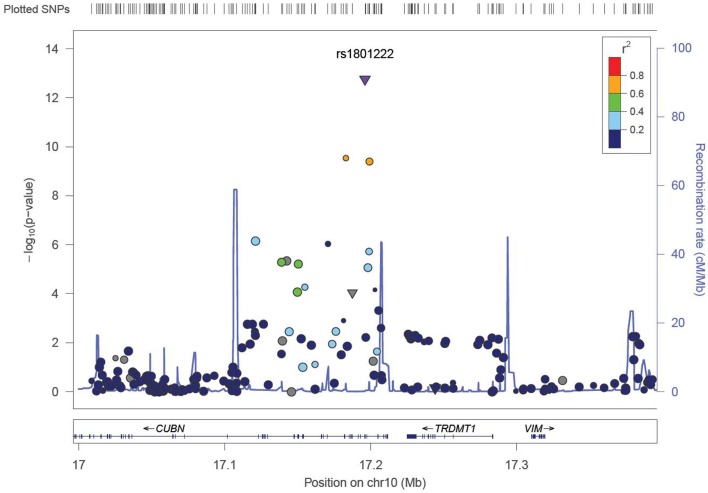
**LocusZoom (49) association plot for single SNP associations with Vitamin B_12_ at the *CUBN* locus**. The SNP position and -LOG (*P*-value) are plotted on the *X* and *Y* axis, respectively.

**Figure 2:**
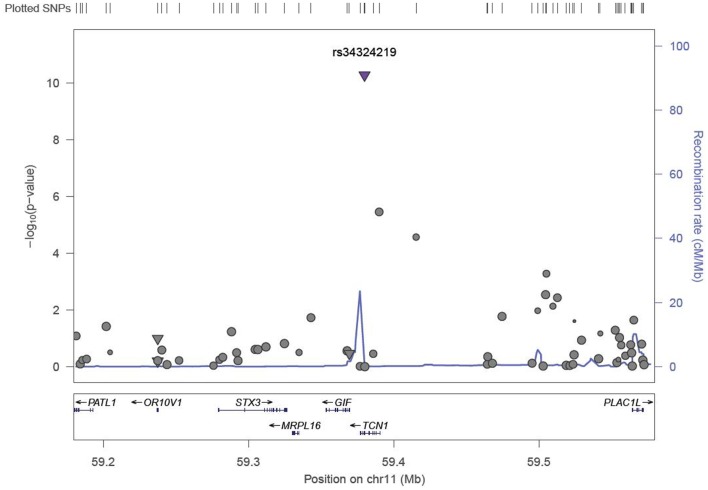
**LocusZoom association plot for single SNP associations with Vitamin B_12_ at the *TCN1* locus**. The SNP position and -LOG (*P*-value) are plotted on the *X* and *Y* axis, respectively.

**Figure 3:**
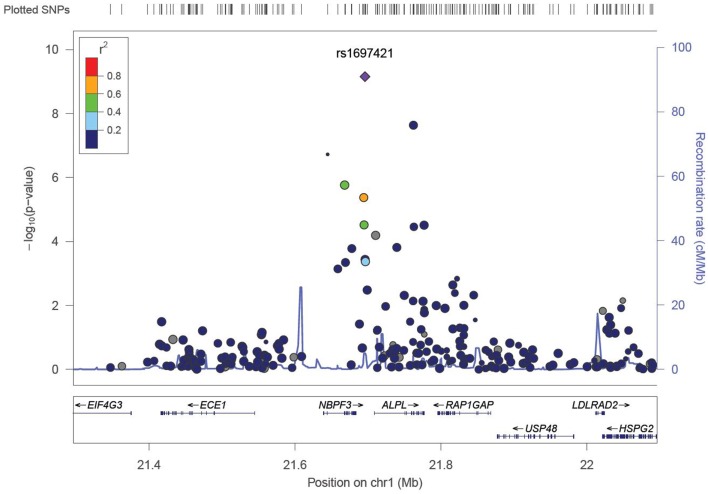
**LocusZoom association plot for single SNP associations with Vitamin B_6_ at the *ALPL* locus**. The SNP position and -LOG (*P*-value) are plotted on the *X* and *Y* axis, respectively.

We identified six associations that meet or exceed genome-wide significance (*P* ≤ 5 × 10^−08^; Table 2; Figure S1 in Supplementary Material). Of these six associations, four were for Vitamin B_12_, while the remaining two were for Vitamin B_6_. The strongest evidence of association was observed for baseline Vitamin B_12_ (*P* = 1.76 × 10^−13^; beta = −0.22) with a non-synonymous SNP (nsSNP), located on chromosome 10 in the *CUBN* gene. Two additional *CUBN* intronic SNPs (Figure 1) were also strongly associated with Vitamin B_12_ (*P* = 2.92 × 10^−10^; beta = −0.19 and 4.11 × 10^−10^; beta = −0.18). A second nsSNP, located on chromosome 11 in the transcobalamin 1 (*TCN1*) gene (Figure 2), was also associated with baseline measures of Vitamin B_12_ (*P* = 5.14 × 10^−11^; beta = −0.29). The two genome-wide significant associations for Vitamin B_6_ measures (Figure 3) were located in the alkaline phosphatase (ALPL), liver/bone/kidney gene region (rs1697421; *P* = 7.06 × 10^−10^, beta = 0.173 and rs1780316; *P* = 2.25 × 10^−08^; beta = −0.325). Although not reaching the genome-wide significance threshold, our GWAS study has identified nine additional SNPs with suggestive (*P* ≤ 10^−07^) evidence of association, two for measures of Vitamin B_6_, six for measures of Vitamin B_12_, and one for measures of folate (Table 2). A cluster of suggestive associations for Vitamin B_12_ was observed on chromosome 19, near the fucosyltransferase 2 (*FUT2)* gene. *P*-Values for the four associated SNPs ranged from 9.33 × 10^−07^ to 2.67 × 10^−07^. Individually, each associated SNP explains only a small amount of the variance (*h*^2^) for each trait (ranging from 0.009 to 0.021).

**Table 2:** **Association results for SNPs with genome-wide (*P* ≤ 5 × 10^−0^^8^) significance or suggestive evidence (*P* ≤ 10^−0^^7^) for association**.

SNP	Chromosome	Location (bp)*	Minor allele	Minor allele frequency	Beta	STAT	Variance explained	*P*-value (bold indicates *P* < 5 × 10^−08^)	Gene (bold indicates novel finding)
**VITAMIN B6**									
rs12118362	1	21644584	A	0.213	0.172	5.228	0.010	1.91 × 10^−07^	***NBPF3***
rs1697421	1	21695879	T	0.471	0.173	6.197	0.015	**7.06 × 10**^−^**^10^**	*ALPL* (nearest)
rs1780316	1	21762222	T	0.060	−0.325	−5.616	0.012	**2.25 × 10**^−^**^08^**	*ALPL*
rs2267739	7	31103422	G	0.153	−0.241	−4.910	0.015	9.92 × 10^−^^07^	***ADCYAP1R1***
**VITAMIN B12**									
rs7893634	10	17121145	A	0.415	0.143	4.972	0.010	7.22 × 10^−^^07^	*CUBN*
rs11254363	10	17170699	C	0.258	0.155	4.918	0.009	9.48 × 10^−^^07^	*CUBN*
rs12261966	10	17183006	A	0.311	−0.185	−6.336	0.015	**2.92 × 10**^−^**^10^**	*CUBN*
rs1801222	10	17196157	A	0.316	−0.218	−7.419	0.021	**1.76 × 10**^−^**^13^**	*CUBN*
rs11254375	10	17199198	G	0.312	−0.184	−6.282	0.015	**4.11 × 10**^−^**^10^**	*CUBN*
rs34324219	11	59379954	A	0.104	−0.292	−6.604	0.016	**5.14 × 10**^−^**^11^**	*TCN1*
SNP19-53897957^⇓^	19	53897957	C	0.483	0.142	5.162	0.010	2.69 × 10^−^^07^	*FUT2*
rs516246	19	53897984	A	0.482	0.142	5.164	0.010	2.67 × 10^−^^07^	*FUT2*
rs492602	19	53898229	C	0.482	0.143	5.193	0.010	2.29 × 10^−^^07^	*FUT2*
rs2287921	19	53920084	C	0.471	0.140	4.921	0.010	9.33 × 10^−^^07^	***RASIP1***
**FOLATE**									
rs12611820	2	2462633	C	0.245	−0.169	−4.913	0.011	9.75 × 10^−^^07^	***MYT1L*** (nearest)

* Based on hg18

^⇓^Corresponds to SNP rs516316

In addition to our genome-wide analyses, we performed a look-up of 16 SNPs previously associated with measures of vitamin B_6_ (*n* = 1) (22), vitamin B_12_ (*n* = 12) (22–24, 31), and folate (*n* = 3) (22, 23, 25). Of the 16 SNPs previously reported in the literature, genotype data were available for 10 of the reported SNPs, while surrogate SNPs in high linkage disequilibrium (LD) (*r*^2^ > 0.9) or the most significant SNP within 100 kb of the reported SNP were reported for the remaining six SNPs. Accounting for multiple testing (*P* = 0.05/16), five of the 16 SNPs, located in *ALPL, MS4A3, TCN1, CUBN*, and *FUT2*, were successfully replicated (*P* ≤ 0.003) in our study (Table 3; Table S1 in Supplementary Material). For comparison, association results for the untransformed B vitamin measures are reported in Table S2 in Supplementary Material.

**Table 3:** **Significant associations of 16 previously reported associations of Vitamin B_6_, Vitamin B_12_, and folate**.

SNP	Chromosome	Gene	Literature *P*-value	Reference	Surrogate SNP	VISP *P*-value
**VITAMIN B6**						
rs1256335	1	*ALPL*	1.40 × 10^−^^15^	(22)	–	3.41 × 10^−^^05^
**VITAMIN B12**						
rs1801222	10	*CUBN*	2.87 × 10^−^^09^	(22)	–	1.76 × 10^−^^13^
rs526934	11	*TCN1*	2.25 × 10^−^^10^	(22)	–	3.38 × 10^−^^06^
rs2298585	11	*MS4A3*	2.64 × 10^−^^15^	(24)	rs7929589	8.67 × 10^−^^04^
rs1047781	19	*FUT2*	3.62 × 10^−^^36^	(24)	rs516246	2.67 × 10^−^^07^

## Discussion

We performed a GWAS for measures of Vitamin B_12_, Vitamin B_6_, and folate by evaluating 737,081 SNPs in 2,100 participants from the Vitamin Intervention for Stroke Prevention (VISP) clinical trial. We observed six associations that reached genome-wide significance (*P* ≤ 10^−08^), an additional nine SNPs with suggestive (*P* ≤ 10^−07^) evidence of association, while replicating five previously reported SNP associations. The most convincing associations were observed for measures of Vitamin B12 at the *CUBN* and *TCN1* loci and the *ALPL* locus for measures of Vitamin B6. Although we did not observe any genome-wide significant associations for folate, we did detect suggestive evidence for association (*P* = 9.75 × 10^−07^) near the *MYT1L* gene, located on chromosome 2. Interestingly, genetic variations at this locus have been associated with depression (32) and schizophrenia (33–35). This locus may help explain the recent data positively correlating serum folate levels with cognitive test scores in children (36); suggesting further evaluation of the effects of folate levels in the elderly are warranted.

The most robust associations for Vitamin B_12_ levels were observed at the *CUBN, FUT2*, and *TCN1* loci (Table 2). A cluster of five SNPs spanning the *CUBN* gene provided evidence for association with Vitamin B_12_ measures (*P*-values ranging from 9.48 × 10^−07^ to 1.75 × 10^−13^). The most significantly associated SNP in this region, rs1801222, was a non-synonymous variant resulting in a missense mutation, Phenylalanine to Serine. These results were not surprising considering rs1801222 was previously associated with Vitamin B_12_ measures (22) and the protein expressed by *CUBN* forms a receptor complex responsible for Vitamin B_12_ internalization in the ileum (37). Furthermore, genetic variants in *CUBN* are responsible for the Finnish type of megaloblastic anemia-1 (38) in humans and more broadly for IGS in canines as well (39, 40). A second cluster of suggestive associations near *FUT2* gene were consistent with previously reported associations in this region (24, 25, 41).

A second missense mutation (rs34324219), located in the Vitamin B_12_ binding protein, *TCN1* gene was associated with baseline measures of Vitamin B_12_ (*P* = 5.148 × 10^−11^). The nsSNP, rs34324219, results in an Aspartic acid to Tyrosine substitution and represents the second most significant association in our study. In the same VISP population, our group previously detected associations between genetic variants of the related gene, *TCN2*, and recurrent stroke risk (42). Although *TCN1* is a logical candidate gene influencing Vitamin B_12_ measures in this region, associations with variants in the nearby (~200 kb) *MS4A3* gene (24) suggest that multiple genes in this region may impact Vitamin B_12_ levels. In an attempt to replicate the associations observed in *MS4A3* by Lin et al. (24) (rs2298585), we detected modest evidence of association for the surrogate SNP, rs7929589 (*r*^2^ = 0.39; *P* = 8.67 × 10^−04^; Table 3). The protein encoded by *MS4A3* has been proposed to function as a hematopoietic cell cycle regulator (43), another potential link to the anemia observed in individuals with Vitamin B_12_ deficiency (44).

For measures of Vitamin B_6_, associations at the ALPL locus were most robust. Two variants at this locus reached genome-wide significance (rs1697421; *P* = 7.06 × 10^−10^ and rs1780316; *P* = 2.25 × 10^−08^). GWAS associations for variants near *ALPL* have been reported for Vitamin B_6_ (22). In addition, this region also harbors GWAS associations with traits ranging from TNFα response in patients with rheumatoid arthritis (45) to hematologic traits (46). While the physiological function of ALPLs are unknown, and no direct correlations have been made between *ALPL* variants and cognitive function, tissue non-specific ALPL is increased in Alzheimer’s disease patients (47). Furthermore, Alzheimer’s disease patients have an increased risk of suffering a stroke (48).

The data were collected as part of a randomized clinical trial is a systematic and standardized fashion, which is a major strength of the study. VISP used centralized laboratory analysis on all samples that complied with strict quality standards. The study population all had ischemic stroke and had elevated measures of serum homocysteine, which might limit generalizability. However, we replicated a substantial proportion of the previously identified genetic variants from studies using a more “general population.” All participants in the VISP clinical trial were 35 years of age or older and suffered a stroke within 120 days of enrollment. This study population also represents an older group of individuals (mean age 67.2 years) that is most prone to vitamin deficiency and subsequent public health concerns including dementia and stroke. We are unable to make any comparisons in normal, healthy individuals, or assess the relation of such associations on stroke risk and other vascular disorders; however, collectively, our finding may provide some insight into the genetic factors influencing measures of B vitamins, in a vulnerable population. Although some dietary measures were collected as part of the VISP trial, we were not able to incorporate dietary “exposure” as a covariate in our analyses. Thus, we cannot identify gene by environmental interactions.

In summary, we performed a GWAS for measures of Vitamin B_6_, B_12_, and folate observing six genome-wide significant associations, nine suggestive associations, and successfully replicating 5 of 16 SNPs previously reported in the literature. Our study is the first of its kind evaluating genetic contributors for measurements of B vitamins in a stroke population. Additionally, this knowledge could lead to genetic screening approaches, which could identify pre-symptomatic individuals that could benefit from interventions such as enhanced vitamin supplementation prior to clinical manifestations.

## Authors Contribution

Keith L. Keene – performed locus specific analyses, drafted manuscript, and constructed primary tables and figures. Wei-Min Chen – lead VISP statistical analyst, reviewed and edited manuscript. Fang Chen – performed initial GWAS analyses under the supervision of Wei-Min Chen. Stephen R. Williams – assisted with figures and summary statistics, reviewed and edited manuscript. Stacey D. Elkhatib – conducted initial review of literature for GWAS of B vitamin phenotypes, ran analyses of several candidates prior to GWAS data, reviewed and edited manuscript. Fang-Chi Hsu – contributed to the overall GWAS design and the writing of the manuscript. Josyf C. Mychaleckyj – assisted with statistical analyses, reviewed and edited manuscript. Kimberly F. Doheny – generation of GWAS data and QC of GWAS data, reviewed and edited manuscript. Elizabeth W. Pugh – generation of GWAS data and QC of GWAS data, reviewed and edited manuscript. Hua Ling – generation of GWAS data and QC of GWAS data, reviewed and edited manuscript. Cathy C. Laurie – quality control of the VISP dataset, assisted with statistical analysis, reviewed and edited manuscript. Stephanie M. Gogarten – quality control of the VISP dataset, reviewed and edited manuscript. Ebony B. Madden – Program Director for the project and made contributions to the writing of the manuscript. Bradford B. Worrall – Co-Principal investigator on GARNET, contributed to the design and analysis plan for paper, and made contributions to the writing of the manuscript. Michele M. Sale – Co-Principal investigator on GARNET, contributed to the design and analysis plan for paper, and made contributions to the writing of the manuscript.

## Conflict of Interest Statement

The authors declare that the research was conducted in the absence of any commercial or financial relationships that could be construed as a potential conflict of interest.

## Supplementary Material

The Supplementary Material for this article can be found online at http://www.frontiersin.org/Journal/10.3389/fpubh.2014.00112/abstract
