# Supplementary material for: Genetic Associations with Plasma B12, B6, and Folate Levels in an Ischemic Stroke Population from the Vitamin Intervention for Stroke Prevention (VISP) Trial
Source: Front Public Health. 2014 Aug 6;2:112. doi: 10.3389/fpubh.2014.00112 (PMC4123605; doi:10.3389/fpubh.2014.00112)
Supplement: Supplementary file 1 [file Data_Sheet1.DOCX]

**Supplementary Figure 1. Manhattan plots for associations with (a) Vitamin B_6_ (b) Vitamin B_12_, and (c) folate.**

**A.**

**
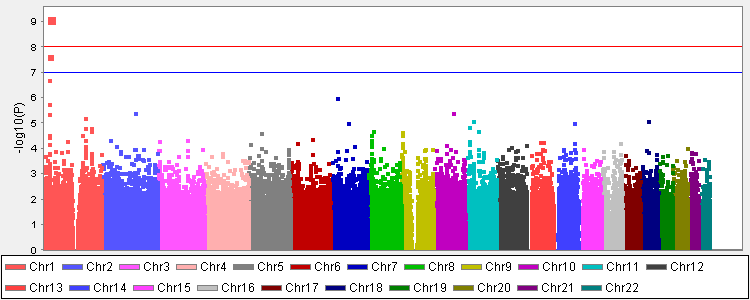
**

**B.**

**
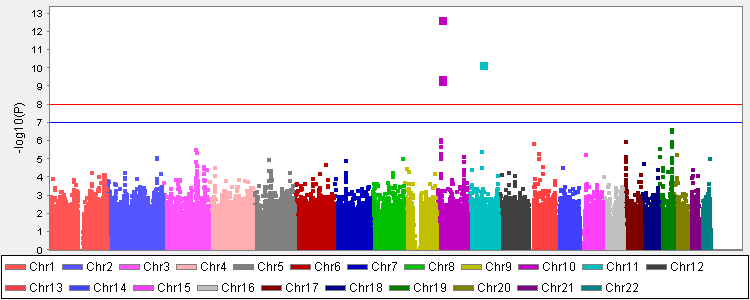
**

**C.**

**
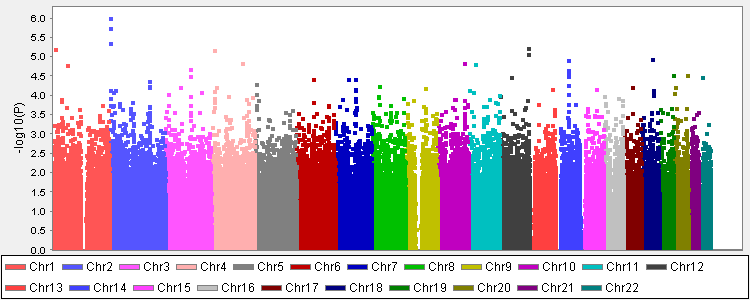
**

**Supplementary Table 1. Replication results of 16 previously reported associations of Vitamin B_6_, Vitamin B_12_, and folate.**

|  | **SNP** | **Chromosome** | **Gene** | **Literature P-Value** | **Reference** | **Surrogate SNP** | **VISP P-Value** |
| --- | --- | --- | --- | --- | --- | --- | --- |
| **Vitamin B6** |  |  |  |  |  |  |  |
|  | rs1256335 | 1 | *ALPL* | 1.40 x 10^-15^ | **[22]** | - | **3.41 x 10^-05^** |
| **Vitamin B12** |  |  |  |  |  |  |  |
|  | rs2270655 | 4 | *MMA* | 2.20 x 10^-13^ | **[23]** | - | 0.108 |
|  | rs10515552 | 5 | *PRELID2* (nearest) | 3.94 x 10^-08^ | **[24]** | - | 0.521 |
|  | rs9473558* | 6 | *MUT* | 4.05 x 10^-08^ | **[22]** | - | 0.0456 |
|  | rs1801222 | 10 | *CUBN* | 2.87 x 10^-09^ | **[22]** | - | **1.76 x 10^-13^** |
|  | rs526934 | 11 | *TCN1* | 2.25 x 10^-10^ | **[22]** | - | **3.38 x 10^-06^** |
|  | rs2298585 | 11 | *MS4A3* | 2.64 x 10^-15^ | **[24]** | rs7929589 | **8.67 x 10^-04^** |
|  | rs41281112 | 13 | *CLYBL* | 9.23 x 10^-10^ | **[24]** | rs7331804 | 0.0138 |
|  | rs3742801 | 14 | *ABCD4* | 1.70 x 10^-13^ | **[23]** | - | 0.614 |
|  | rs3760776 | 19 | *FUT6* | 3.68 x 10^-13^ | **[24]** | - | 0.011 |
|  | rs2336573 | 19 | *CD320* | 8.4 x 10^-59^ | **[23]** | - | 0.022 |
|  | rs1047781 | 19 | *FUT2* | 3.62 x 10^-36^ | **[24]** | rs516246 | **2.67 x 10^-07^** |
|  | rs1131603 | 22 | *TCN2* | 4.9 x 10^-49^ | **[23]** | rs740234 | 0.082 |
| **Folate** |  |  |  |  |  |  |  |
|  | rs1999594 | 1 | *MTHFR* gene region (*KIAA2013* nearest) | 1.12 x 10^-07^ | **[25]** | - | 0.0047 |
|  | rs982393 | 2 | *FIGN* | 8.38 x 10^-08^ | **[22]** | rs4668424 | 0.427 |
|  | rs652197 | 11 | *FOLR3* | 1.4 x 10^-12^ | **[23]** | rs514933 | 0.028 |

* rs9473558 has been merged with rs1141321

**Supplementary Table 2. Association results (untransformed baseline measures) for SNPs with genome-wide (P<5x10^-08^) significance, suggestive evidence (P≤10^-07^), or previously reported SNPs associated with Vitamin B_6_, Vitamin B_12_, and/or folate.**

|  | **SNP** | **Chromosome** | **Location (bp)*** | **Test (Minor) Allele** | **Beta** | **STAT** | **P-Value**  **(bold indicates P<5x10^-08^**) |
| --- | --- | --- | --- | --- | --- | --- | --- |
| **Vitamin B6** |  |  |  |  |  |  |  |
|  | **rs12118362** | 1 | 21644584 | A | 6.435 | 4.366 | 1.34 x 10^-05^ |
|  | rs1697421 | 1 | 21695879 | T | 5.623 | 4.525 | 6.41 x 10^-06^ |
|  | rs1780316 | 1 | 21762222 | T | -9.82 | -3.801 | 1.49 x 10^-04^ |
|  | rs1256335 | 1 | 21762973 | C | -3.946 | -2.604 | 0.009 |
|  | rs12402920 | 1 | 162758778 | T | 7.381 | 4.936 | 8.68 x 10^-07^ |
|  | **rs2267739** | 7 | 31103422 | G | -7.486 | -3.442 | 5.91 x 10^-04^ |
|  | **rs5934651** | X | 9503529 | C | 12.86 | 5.031 | 5.35 x 10^-07^ |
| **Vitamin B12** |  |  |  |  |  |  |  |
|  | rs2270655 | 4 | 146795868 | G | -18.39 | -1.461 | 0.144 |
|  | **rs17539793** | 5 | 141586807 | A | 62.72 | 4.979 | 6.94 x 10^-07^ |
|  | rs10515552 | 5 | 145019024 | G | 6.619 | 0.5268 | 0.598 |
|  | rs9473558 | 6 | 49520392 | A | -4.61 | -0.7621 | 0.446 |
|  | rs7893634 | 10 | 17121145 | A | 21.72 | 3.612 | 3.12 x 10^-04^ |
|  | rs11254363 | 10 | 17170699 | C | 20.08 | 3.052 | 0.002 |
|  | rs12261966 | 10 | 17183006 | A | -26.29 | -4.274 | 2.02 x 10^-05^ |
|  | rs1801222 | 10 | 17196157 | A | -33.96 | -5.525 | 3.75 x 10^-08^ |
|  | rs11254375 | 10 | 17199198 | G | -26.08 | -4.262 | 2.13 x 10^-05^ |
|  | **rs7909327** | 10 | 130044638 | G | 58.62 | 5.632 | 2.04 x 10^-08^ |
|  | rs34324219 | 11 | 59379954 | A | -28.82 | -3.111 | 0.002 |
|  | rs526934 | 11 | 59390069 | G | -20.15 | -3.022 | 0.003 |
|  | rs7929589 | 11 | 59731654 | A | -16.27 | -2.729 | 0.006 |
|  | rs7331804 | 13 | 99307688 | A | 7.846 | 1.204 | 0.229 |
|  | rs3742801 | 14 | 73828759 | A | -2.884 | -0.4765 | 0.634 |
|  | **rs2286305** | 17 | 11776046 | A | 38.19 | 5.158 | 2.75 x 10^-07^ |
|  | rs3760776 | 19 | 5790746 | A | 33.65 | 4.083 | 4.62 x 10^-05^ |
|  | rs2336573 | 19 | 8273709 | A | 38.32 | 3.082 | 0.002 |
|  | SNP19-53897957 ˟ | 19 | 53897957 | C | 20 | 3.472 | 5.28 x 10^-04^ |
|  | rs516246 | 19 | 53897984 | A | 19.88 | 3.452 | 5.67 x 10^-04^ |
|  | rs492602 | 19 | 53898229 | C | 19.91 | 3.452 | 5.67 x 10^-04^ |
|  | rs2287921 | 19 | 53920084 | C | 20.49 | 3.451 | 5.71 x 10^-04^ |
|  | rs740234 | 22 | 29338745 | G | -9.392 | -1.29 | 0.197 |
| **Folate** |  |  |  |  |  |  |  |
|  | rs1999594 | 1 | 11881803 | A | 1.401 | 2.79 | 0.005 |
|  | **rs12611820** | 2 | 2462633 | C | -1.596 | -2.541 | 0.011 |
|  | rs4668424 | 2 | 164162850 | T | -0.6286 | -0.7666 | 0.443 |
|  | **rs7868745** | 9 | 7912071 | G | 3.313 | 5.73 | 1.16 x 10^-08^ |
|  | rs514933 | 11 | 71607855 | G | -0.7531 | -1.454 | 0.146 |
|  | **rs8047337** | 16 | 5063475 | G | 4.545 | 5.314 | 1.19 x 10^-07^ |

* Based on hg18

˟ Corresponds to SNP rs516316

**Novel loci from the VISP study are shown in bold.**
